# The effects of large roughness elements on the in-stream transport and retention of polystyrene microplastics

**DOI:** 10.1038/s41598-023-33436-0

**Published:** 2023-04-21

**Authors:** Usama Ijaz, Abul B. M. Baki, Omar I. Abdul-Aziz, Wenming Zhang, Alan D. Christian

**Affiliations:** 1 Department of Civil and Environmental Engineering, Clarkson University, Potsdam, NY 13699 USA; 2 Department of Civil and Environmental Engineering, West Virginia University, Morgantown, WV 26506-6103 USA; 3 Department of Civil and Environmental Engineering, University of Alberta, Edmonton, AB T6G 1H9 Canada; 4 Department of Biology, Clarkson University, Potsdam, NY 13699 USA

**Keywords:** Civil engineering, Ecology, Freshwater ecology, Restoration ecology

## Abstract

The mechanisms controlling transport and retention of microplastics (MPs) in riverine systems are not understood well. We investigated the impact of large roughness elements (LREs) on in-stream transport and retention of the ubiquitous polystyrene-microplastics (PS-MPs). Scaled experiments were conducted with and without LREs under various shear Reynolds numbers (Re*) in an ecohydraulics flume. Our results, for the first time, demonstrated a clear dependence of the MPs’ velocity on Re* in LREs-dominated channel. Two distinct regimes and thresholds were identified: lower Re* (≤ 15,000) regime corresponding to higher velocities of MPs (${U}_{MPs}^{*}$> 0.45), and higher Re* (> 15,000) to lower ${U}_{MPs}^{*} ($< 0.45). The presence and higher density of LREs increased Re*, decreased ${U}_{MPs}^{*}$, and enhanced the PS-MPs capture. The LREs-generated turbulence kinetic energy (*TKE*) was found to be a good predictor of PS-MPs transport and retention rates, indicating the effectiveness of LREs in retaining PS-MPs in streams and rivers.

## Introduction

Microplastics (MPs) (≤ 5 mm) in the marine environment have been reported and studied for many decades^1,2^. However, MPs gained attention as an emerging contaminant in freshwater systems over the last decade^3^. A significant proportion of marine plastic debris/MPs is assumed to be derived from riverine systems^4^. For example, Meijer et al.^5^ estimated that 1000 rivers globally transport 0.8 − 2.7 million metric tons of plastic litter each year to the ocean. Riverine systems have also been identified as sinks to accumulate or retain MPs^6^. MPs concentration in sediments was found to be 1600 ± 191 particles/kg in Shanghai River, China^7^, as well as 18 to 629 particles/kg in Antua River, Portugal^8^. However, research on riverine MPs is still in its infancy, and MPs’ dynamics in riverine systems are still unknown or unclear^9^.

In riverine systems, MPs are introduced from both point and non-point sources^10^. The watershed processes such as surface runoff, rainfall events, river flow regimes, and flooding may play significant roles on the transport of MPs in the riverine system^11–13^. Vegter et al.^14^ suggested that the in-stream large roughness elements (LREs) such as vegetation and boulders/rocks might influence the retention and transport of MPs, similar to their effects on the deposition of sediment particles. Understanding how MPs are deposited in streams/rivers and transported to the sea is essential for formulating suitable solutions to limit and mitigate the environmental effects of MPs.

The biophysical factors such as the physical/chemical characteristics of MPs, channel morphology, surface roughness (e.g., LREs), and flow rate play significant role in the transport of MPs in riverine environments. A few studies have assessed potential transport and retention characteristics of MP particles in aquatic environments^15–18^. Ballent et al.^15^ estimated the suspension of the MPs with the critical shear stress at which the bed load starts to move, and concluded that 75% of MPs remain in suspension at the same critical stress. Nizzetto et al.^16^ described the theoretical concept for the transport of MPs in the river and explained the retention of MPs was governed by their shape, size, and density. Zhang^19^ conducted a study on transport of MPs in coastal seas and determined that the speed of MPs had been controlled by their physical parameters such as density, size, and shape. Waldschläger and Schüttrumpf^20^ proposed new formulas to describe settling and rise velocities of various MP pellets and fibers with a large variety of shapes, sizes and densities. Sarkar et al.^18^ studied the fate and transport of MPs in the river environments and concluded that stream hydro-morphological characteristics such as the vegetation intensity and boulders density can play significant roles in the settling and transport of MPs. de los Santos et al.^21^ found that denser MPs had been retained more by marine vegetated canopies than less dense MPs, which remain suspended in the water.

Given the limited prior investigations and inadequate understanding, new research is needed to identify the dominant factors (such as the impacts of LREs) controlling the transport and retention of MPs in streams and rivers. Specifically, there is a critical lack of understanding about how the LREs-associated turbulence and various hydraulic parameters (e.g., bed shear stress, bed shear velocity, and turbulent kinetic energy) relate to the transport and retention of MPs in open channels. The main objective of this study is to investigate the influence of in-stream placement of LREs and the role of associated hydraulics on the dynamics (transport/retention) of Polystyrene microplastics (PS-MPs). We evaluate the hypothesis that the flow hydrodynamics of LREs would significantly control the microplastics’ transport and retention in streams and rivers by conducting comprehensive scaled experiments in an open channel.

## Materials and methods

### Experimental setup and scenarios

**Figure 1:**
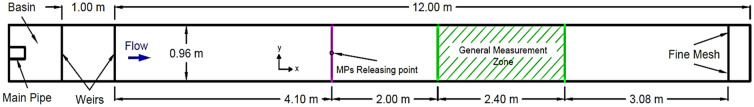
The plan view of the eco-hydraulics flume.

The experiments were performed in the 13 m long, 0.96 m wide, 1.0 m high, recirculating eco-hydraulics flume at Clarkson University. The bed of the flume was composed of a 50-mm thick fine gravel layer with a median diameter of 6.1 mm. These gravels were glued together to avoid potential erosion. The bed slope was *S*_0_ = 0.5%. The longitudinal, transverse, and vertical dimensions of the flume were denoted by *x*, *y*, and *z* directions, respectively. An observation area was defined as a portion of the flume that was about 2.4 m long starting 6.10 m downstream of the flume entrance, to ensure a fully developed turbulent flow (Fig. 1). A Vectrino Plus (Nortek) acoustic Doppler velocimeter (ADV) was used to measure velocity time-series in *x*, *y*, and *z* directions. The ADV was mounted on a carriage in the observation area, which enabled the automatic movement of the ADV in three dimensions (3D) with a resolution of 0.1 mm. Water depth was measured with a point gauge and controlled with a flume tail-gate. The PS-MPs were released from releasing point at flume center line (4.1 m downstream from the start of the flume and 0.01 m below the water surface to avoid surface tension). A special designed screen (1 mm opening size) was positioned at downstream of the flume to capture the released MPs. The distance between the MPs releasing point to the screen is 7.48 m (Fig. 1).

The industrial MPs pellets, made of Polystyrene (PS), used for the experiments, which is especially utilized for packaging material like shockproof containers and food packaging^22^. Bai et al.^23^ found that the most common polymer types detected in rivers are polyethylene (PE) (42%), followed by polypropylene (PP) (30%), and PS (11%), the third most abundantly found MPs in the rivers. The reason behind choosing the PS is their density (1,050 kgm^-3^), slightly more than water, while PE and PS have smaller density than water. The PS-MPs were ordered from Cospheric Laboratory, CA. The dimension of the MPs was 5.00 ± 0.1 mm in diameter. The use of this size has advantage that MPs are more easily identified during the experiment such as with GoPro cameras (GoPro Inc). The settling velocity of PS-MPs (*w*_s_ = 5.2 cm/s) was calculated from experiments performed in a graduated cylinder, which is further supported by the results of Khatmullina and Isachenko^24^, who reported values of 2—18 cm/s for MPs spheres.

Three different experimental scenarios were used in this study (see Table 1): no LREs (A1—A3); (ii) vegetation with disperse vegetation (B1- B4) and vegetation along banks (C1—C4); and (iii) Scattered boulder’s arrangement (D1- D8), under two different with/without tail-gate conditions. The details of all the scenarios are given in the Supplementary Information.

**Table 1:** Summary of the experimental scenarios.

Scenarios	Conditions	Tail-gate	LRE density *λ* (*%)*	Flow rate Q (m^3^/s)	Water depth* H* (*m*)	Flow velocity *U*_avg_ (*m/s*)	Bed shear velocity $u_{*}$ (*m/s*)	Shear Reynolds number Re*
A1	No LRE	Tail-gate	0.0	0.060	0.202	0.22	0.084	16,970
A2	No LRE	No	0.0	0.060	0.076	0.61	0.057	4,311
A3	No LRE	No	0.0	0.075	0.096	0.72	0.063	6,014
B1	Disperse vegetation	Tail-gate	7.0	0.060	0.214	0.22	0.102	21,887
B2	Disperse vegetation	No	7.0	0.060	0.097	0.48	0.068	6,638
B3	Disperse vegetation	Tail-gate	10	0.060	0.221	0.21	0.104	23,097
B4	Disperse vegetation	No	10	0.060	0.124	0.48	0.077	9,617
C1	Vegetation along banks	Tail-gate	5.0	0.060	0.220	0.20	0.104	22,851
C2	Vegetation along banks	No	5.0	0.060	0.107	0.41	0.072	7,804
C3	Vegetation along banks	Tail-gate	10	0.060	0.230	0.19	0.106	24,540
C4	Vegetation along banks	No	10	0.060	0.124	0.30	0.078	9,744
D1	Scattered boulders	No	3.4	0.060	0.099	0.46	0.067	6,647
D2	Scattered boulders	No	3.4	0.075	0.127	0.54	0.077	9,735
D3	Scattered boulders	No	5.4	0.060	0.115	0.44	0.073	8,360
D4	Scattered boulders	No	5.4	0.075	0.141	0.55	0.081	11,386
D5	Scattered boulders	No	8.3	0.060	0.129	0.42	0.076	9,916
D6	Scattered boulders	No	8.3	0.075	0.151	0.50	0.083	12,600

### Data collection

Hydraulic data of the varying scenarios were recorded with ADV with 100 Hz sampling rate for a duration of 180 s. At each measuring location, three-dimensional (3D) velocity time series were recorded at relative depths of *z/H* = 0.05 or 0.10, 0.20, and 0.04 (depending on flow) over the flow depth, here *H* is the reach average water depth and *z* is the vertical distance from the flume bed. Based on the measurement taken and the assumption from Liu et al.^25^, measurement locations were selected 45 cm away from the start of the measurement zone as highlighted in Figure S1 (in Supplementary Information) with red points. The measuring points were selected based on preliminary test measurements to capture the mean flow hydraulics in the vegetation. For boulder scenarios, velocity measurements were taken in the detailed measurement zone (0.72 m × 0.36 m ) over a grid (Figure S2 in Supplementary Information, where boulders were placed in a staggered arrangement throughout the flume (details are available in Golpira et al.^26^). The raw data were processed with the use of WinADV to remove spikes using the method of Goring and Nikora^27^. To eliminate the poor-quality data, signal-to-noise ratio (SNR) and velocity signal correlations (COR) are commonly used. A filtering scheme with an average COR ≤ 70% and average SNR ≤ 15 dB was used to eliminate low quality data from the velocity time series to yield reliable data^28,29^.

The primary method of measuring retention of the PS-MPs was by counting the number of PS-MPs captured by the screen after a given time. i.e., one minute to travel through the flume. After every run, the pump was turned off and PS-MPs were collected throughout the flume to make sure that bed of the flume was ready for the next experiment. One hundred PS-MPs were released from the releasing point and a timer was used to record the time required for the first and final PS-MPs to reach the screen. These times were used to determine the average velocity for the PS-MPs (*U*_*MPs*_) through the flume. For some scenarios (D1-D6), the estimated velocity was verified by tracking the motion of PS-MPs with the help of Adobe After Effect, V.18.0 (Adobe Inc.).

Furthermore, to determine the velocity and transport behavior of PS-MPs in the observation area, two GoPro cameras were used. One GoPro camera was mounted on the exterior left wall of the flume at the midpoint of the observation section and was able to record at a frequency of 50 Hz through a clear plexiglass window, as not to affect any flow. The approximate line of sight for the GoPro camera was 1.0-m length within the flume. For scenarios B3, B4, C3, C4, D5 and D6, an additional GoPro camera was attached on the interior right wall of the flume in the water to cover the observation area.

### Analytical methods

Reach average bed shear stress $\left({\tau }_{o}\right)$ is defined as the force exerted by flowing water against the bed of the channel and is calculated as;

1 $${\tau }_{o}= \rho g{R}_{h}{S}_{0}$$

where, *ρ* is the water density, *g* is the acceleration due the gravity, and *R*_h_ is the hydraulic radius for no LREs and volumetric hydraulic radius having LREs^30^. Equation (1) is not meaningful to apply for a bed covered with LREs, where a portion of the stress is borne by the LREs. Therefore, additional parameters that have influence on the drag force such as drag coefficient ($${C}_{d})$$, height of the individual LRE (*l*), and diameter of individual LRE (*d*)$$,$$ were used to calculate the effective $${\tau }_{o}$$ for the LREs dominated scenarios. The equation for $${\tau }_{o}$$ in the LRES dominated bed was modified as^31^ as follows:

2 $${\tau }_{o}= \rho g{R}_{h}{S}_{0}- 1/2\rho H{C}_{D}ld{U}_{avg}^{2}$$

Herein, the *C*_*D*_ for boulders is determined as $1.787 ({\frac{H}{d})}^{-2.16}$ following Baki et al.^30^. *U*_*avg*_ is the depth-averaged flow velocity*.* ${R}_{h}$ depends on certain parameters like *H*, λ and ${l}^{*}$, where ${l}^{*}$ is the ratio of boulder height to average flow depth, *l/H*. The ${R}_{h}$ for LRE was calculated with Eq. (3):^30^

3 $${R}_{h}=H(1-\frac{2}{3}\mathrm{ \lambda}{l}^{*})$$

The average bed shear velocity ${u}_{*}$ and the shear Reynolds Number, $R_{e}^{*}$, were calculated following Eq. (4) and Eq. (5) as:

4 $$u_{*} = {\sqrt {\tau_{0} } }/\rho$$

5 $$Re^{*} = \frac{{\rho Hu_{*} }}{\mu }$$

where, *μ* is the dynamic viscosity of water and *H* is assumed as the thickness of the turbulent boundary layer. $R_{e}^{*}$ is a suitable function to define the relation of the effective force of the flow to the resistance of a particle in the near-bed zone^32^.

The instantaneous velocity measured by ADV was decomposed into time-averaged (*u, v,* and *w*) and fluctuating (*u’*, *v’* and *w’*) velocity components in *x*, *y*, and *z* directions. The depth-averaged velocity (*U*_*avg*_) between the LREs is the mean of the time-average velocity (*u)* and was computed as $U_{avg} = \frac{1}{n}\mathop \sum \limits_{i = 1}^{n} u$, where *n* is the representative of measurements points in the observation zone. Likewise, the depth-averaged turbulent kinetic energy (*K*) for the bare bed was determined by $\left( {TKE} \right)_{avg} = \frac{1}{n}\mathop \sum \limits_{i = 1}^{n} \left( {TKE} \right)$, where *TKE* is turbulent kinetic energy at any point. In a channel with LREs, both the bed-generated turbulence and the vegetation-generated turbulence contribute to the average turbulent kinetic energy (*K*) and can be estimated as^33^:

6 $$TKE = \frac{1}{2}(u_{rms}^{^{\prime}2} + v_{rms}^{^{\prime}2} + w_{rms}^{^{\prime}2} )$$

where $u_{rms}^{^{\prime}}$, $v_{rms}^{^{\prime}}$, $w_{rms}^{^{\prime}}$ is the root mean square velocity in the streamwise, transverse, and vertical directions, respectively.

### Rouse number

Rouse number (*R*_*o*_) was calculated to quantify the relationship between particle settling velocity (*w*_*s*_) and the upward velocity generated by turbulent eddies. *R*_*o*_ is commonly used to estimate the mode of the sediment transports in turbulent flows with large Reynolds number. The *R*_*o*_ leads to the critical condition for sediment transports mode in flowing water and written as^34^:

7 $$R_{o} = \frac{{w_{s} }}{{\kappa u_{*} }}$$

where $\kappa { }$ = von Karman’s constant (= 0.4 for clear water). In turbulent flow, for large *R*_*o*_ > 2.5, the sediments are transported as a bed load; for 1.20 < *R*_*o*_ < 2.5 and 0.8 < *R*_*o*_ < 1.20, 50% and 100% of the sediments move like suspended load, respectively; and for small *R*_*o*_ < 0.8, the sediments are transported in the wash load mode^35^.

### Retention coefficient

The number of particles retained in the given reach can be defined by the retention efficiency of a channel. The negative exponential model is mostly used to determine the retention rate or settlement behavior of particles^36^.

8 $$n_{x} = n_{i} *e^{{ - l{\text{k}}}}$$

Herein, $n_{x}$ is the number of particles entrained in the flow at the given distance ($l$), $n_{i}$ is the number of particles released in the flow, $l$ is the distance of from the release point to the collecting point, and *k* is the retention coefficient. The larger the value of the *k*, the larger the number of PS-MPs retained in the given channel reach.

## Results and discussion

### MPs dynamics

**Figure 2:**
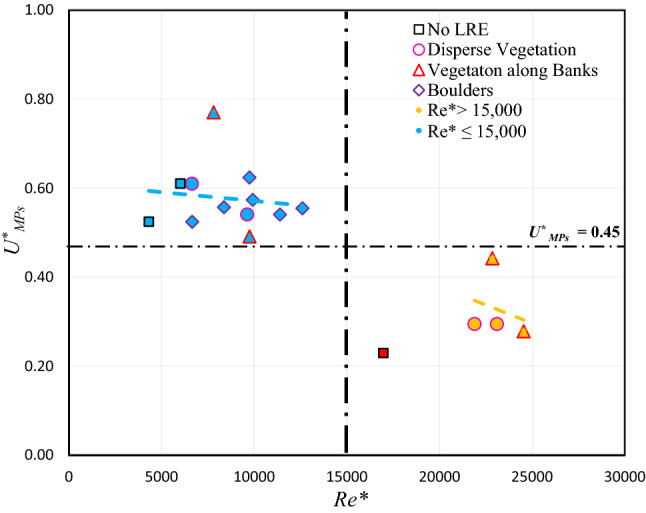
The relationship between shear Reynolds number (Re*) and dimensionless microplastics velocity ($U_{MPs}^{*}$). (The red data point is for no LREs scenario and is not considered in the analysis).

The movement of MPs in the riverine systems is complex because of the complex hydrodynamic and morphodynamic conditions. The in-stream transport of MPs is investigated based on the shear Reynolds number (Re*). The experimental scenarios resulted in two distinct categories of Re*: lower Re* ≤ 15,000 implies lower bed shear stress and lower water depth, and higher Re*  > 15,000 implies higher bed shear stress having higher water depth due to regulated conditions using tail-gate (Table 1). The higher values of Re* belongs to the scenarios having tail-gate (i.e., regulated river) and are much higher than no tail-gate scenarios (i.e., non-regulated natural river). Moreover, the Re* values for tail-gate scenarios B1, B3 and C1, C3 are greater than that in scenario A1 that has no LREs. For Re* ≤ 15,000, the dimensionless velocity of PS-MPs, $U_{MPs}^{*}$ (= *U*_*MPs*_*/U*_*No-LRE*_ > 0.45), decreases with increasing Re* (Fig. 2). The Re* increases with increasing the density of LREs as a result of increasing turbulent shear stress (Table 1). The same trend, as expected, was observed for Re* > 15,000, $U_{MPs}^{*}$ (< 0.45) decreases with increasing Re*. The literature reported a similar pattern for the LREs channel, i.e., a gradual increase in the boulder concentration increase in near-bed shear stress^37–39^. To summarize, at a lower Re*, the rate of change of $U_{MPs}^{*}$ with Re* is slower, as the dense LREs suppress the flow velocity, than that at higher Re*. It can be posited that there exists a clear dependence of the MPs velocity on Re*, supporting the study hypothesis that turbulence (i.e., turbulent shear stress) may affect the transport rate of MPs in the riverine system.

In turbulent flow, the high-density MPs can transport over long distances in suspension or settle down at the river bottom after a certain distance^6^, depending on MPs properties and biophysical factors of the water environment. For each experimental scenario, the Rouse number (*R*_*o*_) is estimated to understand the stream transport mode of MPs (Table 2). The estimated *R*_o_ ranges between 1.22 and 2.29, which reveals that 50% of PS-MPs are transported like suspended load^34^ by the upward flux of turbulence generated at the channel bed. The rest of the PS-MPs as bed load are kept in motion (rolling and sliding), partly supported by the turbulence of the flow and partly by the shear stress acting on the bed. The above phenomena can be verified by Francalanci et al.^40^, in which they concluded that PS-MPs have low-density polymer travel at the sub-surface of the water. Furthermore, Scherer et al.^41^ found 5.57 MPs particles/m^3^ in the water column of the German River Elbe, although these concentrations were about 600,000-fold lower than those in the sediments.

**Table 2:** Summary of key experimental results-

Code	Tail-gate	MPs velocity *U*_*MPs*_ (*m/s*)	Rouse number (*R*_*o*_)	Retention coefficient *k* (*1/m*)	PS-MPs pass (*%*)
A1	Tail-gate	0.14	1.55	0.00970	93
A2	No	0.32	2.29	0.00000	100
A3	No	0.44	2.08	0.00000	100
B1	Tail-gate	0.18	1.27	0.01559	89
B2	No	0.33	1.89	0.00134	99
B3	Tail-gate	0.18	1.25	0.01863	87
B4	No	0.33	1.67	0.00827	94
C1	Tail-gate	0.27	1.25	0.00546	96
C2	No	0.47	1.79	0.00000	100
C3	Tail-gate	0.17	1.22	0.00827	94
C4	No	0.30	1.67	0.00407	97
D1	No	0.32	1.94	0.00000	100
D2	No	0.45	1.69	0.00451	98
D3	No	0.34	1.78	0.00912	96
D4	No	0.39	1.60	0.00912	96
D5	No	0.35	1.69	0.02604	89
D6	No	0.40	1.56	0.02107	91

**Figure 3:**
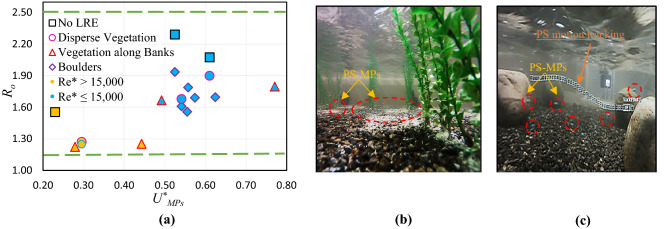
(**a**) The relationship of Rouse number (Ro) and dimensionless microplastics velocity ($U_{MPs}^{*}$), with yellow highlighted dots (Re* > 15,000); (**b**) observations of PS-MPs’ movement/ settlement in vegetation zone (B2); and (**c**) observations of PS-MPs’ movement/ settlement in boulder zone (D5) (the motion of PS-MPs highlighted with the sequence of squares).

Figure 3(a) shows the speed of PS-MPs with respect to the *R*_o_ for all the scenarios. The scenarios (A1, B1, B3, C1, and C3) having tail-gate, have smaller *R*_*o*_ values (< 1.56) and slower transport speed ($U_{MPs}^{*}$ < 0.45), because of relatively higher values of $u_{*}$, where the lift velocity of PS-MPs due to $u_{*}$ is larger than the deposition rate of the particles. For the rest of the scenarios without tail-gate, *R*_*o*_ values are relatively larger (≥ 1.56), and the transport speed is higher ($U_{MPs}^{*}$ > 0.45) than those in tail-gate scenarios, where the lift velocity of PS-MPs is about the particle settling velocity. The movement/settlement of PS-MPs over the bed within the vegetation zone (scenarios B2) and boulders zone (scenario D5) could be visualized using Fig. 3(b) and (c), respectively. Figure 3 illustrates the rolling and saltation processes over the bed (as discussed above) as the PS-MPs move forward. Figure 3(c) shows the tracking motion of PS-MPs in scenario D5. The particle tracking methods show that PS-MPs follow some path lines, then touch the ground after some instant.

**Figure 4:**
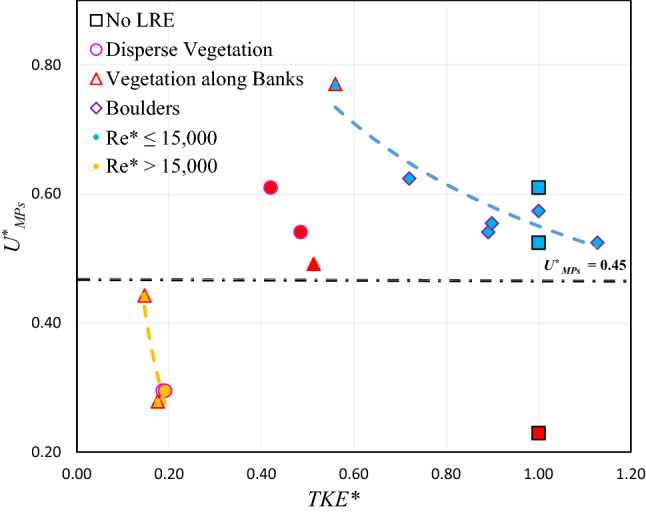
Shows the relationship between dimensionless microplastics velocity ($U_{MPs}^{*}$) and dimensionless turbulence kinetic energy ($K_{e}^{*}$). (The red data points are considered outliers and not considered in the nonlinear regression analysis).

For the LREs dominated channel, some studies^38,42^ demonstrated that the transport of sediment is more closely correlated with *TKE* than with the bed shear stress. Similar to the sediment particle, the dependence of the in-stream velocity of PS-MPs ($U_{MPs}^{*}$) on *TKE (*$TKE^{*}$ = *TKE/TKE*_*No-LRE*_ ) is shown in Fig. 4. $U_{MPs}^{*}$ and $TKE^{*}$ showed a strong negative relationship for distinct categories of scenarios (low Re* for non-regulated and high Re*for regulated conditions), where the squared Pearson correlation coefficient (*r*^2^) is greater than 0.80. The power relationships Eqs. (9) and (10) between $U_{MPs}^{*}$ and $TKE^{*}$ for Re* ≤ 15,000 and Re* > 15,000, respectively, are as follows:

9 $$U_{MPs}^{*} = 0.5504 \left( {TKE^{*} } \right)^{ - 0.498} \quad \left( {r^{2} = \, 0.{81}} \right)$$

10 $$U_{MPs}^{*} = 0.0109 \left( {TKE^{*} } \right)^{ - 1.904} \quad \left( {r^{2} = \, 0.{89}} \right)$$

The rate of change in $U_{MPs}^{*}$ and $TKE^{*}$ is faster (exponent = 1.904) in scenarios for and Re* > 15,000 than the scenarios (exponent = 0.498) for Re* ≤ 15,000.

The negative relationships between $U_{MPs}^{*}$ and $TKE^{*}$, $U_{MPs}^{*}$ decreases with increasing $TKE^{*}$ value, suggests a meaningful physical phenomenon. The factors which dampen MPs velocity in flowing waters can be interpreted according to the *TKE* associated with the LREs. This means that the escalation of turbulence (i.e., TEK) in LREs leads to the reduction of PS-MPs velocity, further supporting the hypothesis. Changes in *TKE* with increasing LREs density reflect the competing effects of the reduced flow velocity^43^. However, a nonlinear response was reported in which turbulence levels initially increase with increasing density of LREs but decrease as the density increases further^39,43^. Similarly, the measured *TKE* in all experimental scenarios suggested a nonlinear response/trend between turbulence and density of LREs. Therefore, further experimental scenarios having various densities of LREs could be used to investigate the effects of *TKE* on the velocity of MPs.

### MPs retention

The retention efficiency of a channel (i.e. the proportion of particles retained within a given reach) describes settlement patterns of particles^36^. In general, the presence of LREs increases the retention (i.e., decrease in percentage of pass) of PS-MPs compared to the bare bed flume, as expected (Table 2). Ehrman and Lamberti^44^ proposed that the degree of retention is related to the number of retentive structures per reach length.

**Figure 5:**
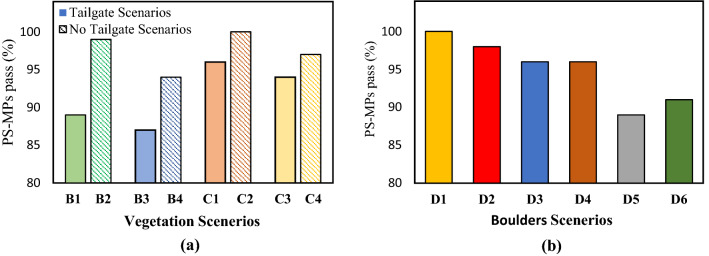
The percentage pass of PS-MPs for different scenarios (**a**) vegetation scenarios and (**b**) boulders scenarios.

A comparision between boulders and vegetation scenerios (No tailgate) was conducted in Fig. 5, and it was observed that boulders are more effective in retaining of PS-MPs then vegetation scenerios with same density (< 8.3%) and same flow condition (0.060 m^3^/s). In vegetation scenerios, all the PS-MPs pass and no retention was noticed for the vegetation denisty lower than 8.3%. On the other hand, some percentage of PS-MPs retained in the boulder scenerios for the boulder density (5.4%) in the observation zone. Furthermore, it was noticed that high vegetation density scenerios (B3, B4, C3, C4) tend to retain more PS-MPs then low density scenerios (B1, B2, C1, C3) with and without tailgate (Fig. 5 (a)). However, no specific trend was found in vegetation scenerios. In boulders scenerios, the PS-MPs percentage pass increases with the increase in boulders density (Fig. 5 (b)). For flow condition 0.060 m^3^/s, the rentention is twice as the boulder density is increased from 3.4% to 8.3%. The same trend was followed by the boulders scenerios having a flow of 0.075 m^3^/s (i.e. more retention was notice by the large boulders density scenerios).

**Figure 6:**
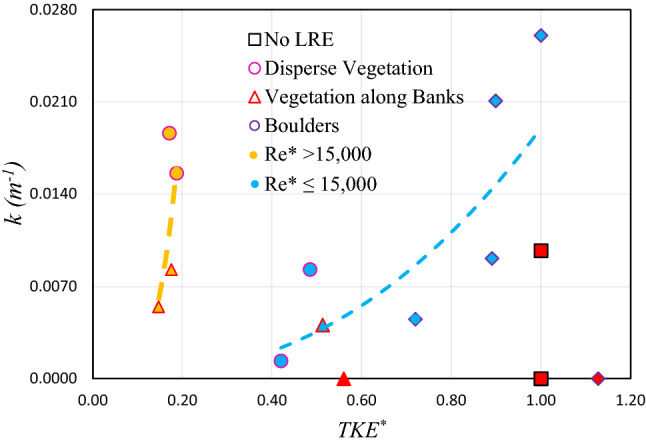
The relationship between dimensionless turbulence kinetic energy ($TKE^{*}$) and retention coefficient (k) for low and high shear Reynolds number ($R_{e}^{*}$) series of experiment. (The red data points are considered outliers and not considered in the nonlinear regression analysis).

As the *TKE* is significantly correlated with the in-stream velocity of PS-MPs in this study (as discussed above), it is important to have a relationship between retention efficiency (*k*) and $TKE^{*}$ (Fig. 6). As expedted, positive correlation was observed between $TKE^{*}$ and *k* values for both categories (for Re* ≤ 15,000 and Re* > 15,000) of scenarios. Where, the value of the *k* increases as $TKE^{*}$ increases. For Re* ≤ 15,000, *k* increases from 0.0013 (1/m) to 0.0260 (1/m) (about 20 times) with increasing $TKE^{*}$ from 0.4197 to 1.0, and for Re* > 15,000, *k* increases from 0.0055 (1/m) to 0.0186 (1/m) (about 3 times) with increasing $TKE^{*}$ from 0.1465 to 0.1911, suggesting a significant influence of *TKE* on the PS-MPs retention, as well as supporting the hypothesis that *TKE* affect the retention of MPs in the riverine system. This mean that the retention of PS-MPs increases with the increase in turbulence; the higher density of LRE (λ ) resulted strong *TKE* due to the generation of stem wake turbulence^45^. Therefore, λ can be considered another factor that can directly influence the retention of PS-MPs^21^. However, finding an optimum LREs concentration, at which the retention is maximized, requires testing various LREs concentrations and was not achieved in this study.

The relationships can be expressed as power Eqs. (11) and (12) for Re* > 15,000 and ≤ 15,000, respectively, a comparison of different scenarios showed that PS-MPs were retained more proportionately in tail-gate scenarios as compared to the scenarios without tail-gate. For Re* ≤ 15,000, the PS-MPs retained more slowly than the scenarios for Re* > 15,000. The *r*^2^ between $k$ and $TKE^{*}$ for Re* ≤ 15,000 is not good (*r*^*2*^ = 0.35) however for Re* > 15,000, it shows a decent correlation (*r*^*2*^ = 0.73), which shows a higher dependency of $k$ on $TKE^{*}$.

11 $$k{ } = { }13.418{ }\left( {TKE^{*} } \right) ^{4.0125} \quad \left( {r^{2} = \, 0.{35}} \right)$$

12 $$k{ } = { }0.019{ }\left( {TKE^{*} } \right) ^{2.4116} \quad \left( {r^{2} = \, 0.{73}} \right)$$

**Figure 7:**
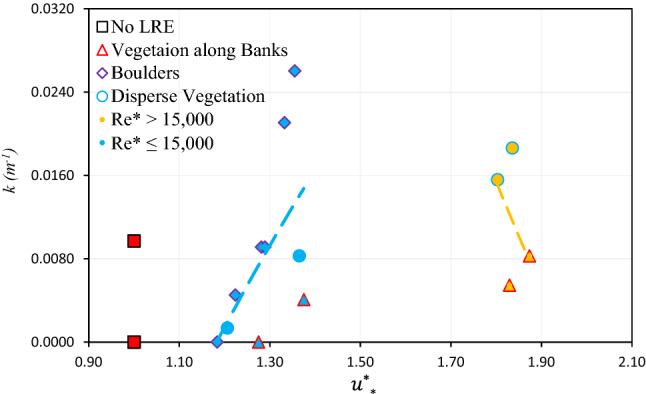
The relationship between dimensionless bed shear velocity $\left( {u_{*}^{*} } \right)$ and retention coefficient (k). (The red data points are considered outliers and not considered in the nonlinear regression analysis).

This study also examined the influence of dimensionless shear velocity $$u_{* }^{*} ( = u_{*} /u_{*}^{no - LRE}$$) on the retention coefficient (*k*) of PS-MPs as shown in the Fig. 7. The result indicates almost two opposite relationships between retention coefficient ($$k)$$ *and* $$u_{* }^{*}$$ based on two distinct categories (low and high Re*) of scenarios.

Similar to *TKE*, the bed shear velocity, $u_{*}$, in the LREs dominated channel could be a good predictor of MPs retention rate, as there is a clear dependence of the MPs velocity on Re* (as discussed above). For Re* ≤ 15,000, the relationship between dimensionless bed shear velocity $u_{*}^{*}$ and the *k* is positive (Fig. 7). Where, *k* increases from 0.0013 (1/m) to 0.0260 (1/m) (about 20 times) with increasing $u_{*}^{*}$ from 1.206 to 1.355, suggesting that the shear stress generated by the bed significantly increased the PS-MPs retention. This justified that the increased $u_{*}$ within the LRE captured more PS-MPs in the study reach during the experiments were through direct interception, diffusional and deposition^31^. *r*^2^ value between $u_{*}^{*}$ and *k* is low (= 0.34) and the following power expression can be obtained:

13 $$k = { }0.0979{\text{ ln}}\left( {u_{*}^{*} } \right) + 0.0164\quad \left( {{\text{r}}^{{2}} = \, 0.{34}} \right)$$

Nevertheless, for the scenarios of Re* > 15,000, the *k* increases sharply with a decrease in $u_{*}^{*}$ (Fig. 7), though the trend is not clear due the scatter data sets. Where the squared Pearson correlation coefficient (*r*^2^) is equal to 0.24. The power relationships Eq. (14) between $u_{*}^{*}$ and *k* for Re*  > 15,000 is as follows:

14 $$k = - 0.19\ln \left( {u_{*}^{*} } \right) + 0.1273\quad \left( {r^{2} = \, 0.{24}} \right)$$

The squared Pearson correlation coefficient (*r*^2^) between $u_{*}^{*}$ and *k* (< 0.35) is lower than the *r*^2^ between $TKE^{*}$ and *k* (≥ 0.35), suggesting that *TKE* is a better predictor of PS-MPs dynamics. Yang and Nepf^41^ also found that *TKE* generated in the vegetated channel is a better metric for predicting the number of sediment grains in motion than flow velocity.

The results of this study confirm our hypothesis that LREs-generated hydraulic parameters (e.g., Re*, *R*_0,_ $u_{*}^{*}$, *TKE* ) significantly control the transport and retention of MPs in streams and rivers. However, results of this study should be interpreted or used with some caveats. First, a single type and size of MPs were used in this study: PS-MPs having a density of 1,050 kgm^-3^. Second, the channel bed was not movable, which resulted in simplifying the in-situ conditions; however, variation in local hydraulics and substrate compositions are expected in a mobile bed around LREs in an open channel. Third, this study is applicable to LREs with a dimensionless density equal to or less than 10%; this is the upper limit through which the estimated equations would work best for PS-MPs characterization and transport.

The current study presents the results as the necessary primary and pivotal stage for future research to achieve an overall robust understanding, characterization, and parameterizations of in-stream MPs transport and retention processes by varying the characteristics of MPs (e.g., type, size, shape, and density) — as well as that of bed substrates, large-roughness elements, and river hydrodynamics. Future studies should also investigate the impact of biological factors on the dynamics of MPs. The data and findings of the current study may be utilized to develop a process-based model to reliably predict the in-stream dynamics of PS-MPs. However, developing an acceptable process-based model of MPs transport and retention is a significant undertaking that merits a separate study. Overall, the findings of our study would serve as a basis for further experimental, numerical, and field studies on the dynamics of MPs in streams and rivers.

## Conclusions

We investigated the influence of in-stream placement of LREs and the role of associated hydraulics on the transport and retention of MPs in an open channel. This study, for the first time, demonstrated a clear dependence of the MPs’ velocity on Re* in LREs-dominated channel. Two distinct regimes and thresholds were identified: lower Re* (≤ 15,000) regime corresponding to higher velocities of MPs ($U_{MPs}^{*}$ > 0.45), and higher Re* (> 15,000) to lower $U_{MPs}^{*}$ (< 0.45). The presence and higher density of LREs increased Re*, decreased $U_{MPs}^{*}$, and enhanced the PS-MPs capture. Another new finding is that the LREs-generated *TKE* was a good predictor of PS-MPs transport and retention rates. The retention coefficient increased with the increase in the average turbulence kinetic energy, indicating the effectiveness of LREs in retaining PS-MPs in streams and rivers. Empirical relationships were developed to predict the velocity and retention coefficient of PS-MPs from *TKE*; these equations can ultimately be helpful and practical for the prediction of in-stream transport/retention of PS-MPs. Furthermore, our study, based on analysis of data for PS-MPs, indicates for the first time that the transport mode of MPs in LRE-dominated streams may essentially be similar to that of sediments retention and transport reported in previous studies.

## Supplementary Information

Supplementary Information.

## Data Availability

The datasets generated during and/or analyzed during the current study are available from the corresponding author on reasonable request.
