# Supplementary material for: The effects of large roughness elements on the in-stream transport and retention of polystyrene microplastics
Source: Sci Rep. 2023 Apr 21;13:6522. doi: 10.1038/s41598-023-33436-0 (PMC10121686; doi:10.1038/s41598-023-33436-0)
Supplement: Supplementary file 1 — Supplementary Information. [file 41598_2023_33436_MOESM1_ESM.docx]

***Supplementary Information***

**S1. Experimental Scenarios**

*S1.1 No LRE scenarios*

A1-A3 scenarios were run without LREs under two flow scenarios (0.075 m^3^/s and 0.060 m^3^/s), including tail-gate (A1) as well as no tail-gate scenarios (A2 and A3). These experiments provided a baseline or reference conditions for how the boulders and vegetation impact the transport of the PS-MPs (Figure S1).

*S1.2 Vegetation scenarios*

For the vegetation scenarios, special types of artificial aquarium plants AQUASCAPERS®, USA, type Anacharis (*Egeria densa*)^1^ with 24.5 cm of stem height and 2.7 cm of stem width were used. The stems were wired with the help of 0.88 mm galvanized steel Hillman^TM^ wire for rigidness and glued in the measurement area of 100 cm in length and 96 cm in width.

A total of eight scenarios were designed. Figure S1 illustrates the plan of all the vegetation scenarios with green squares and rectangular bars show vegetation patches/strips and red points shows the hydraulics measuring points. The first four scenarios (B1- B4) with disperse vegetation patches (11 cm^2^) having densities (ʎ*)* = 7% and 10% in the observation section with *d/s* = 0.20 to 0.49, where *d* is the equivalent diameter of the vegetation patches and *s* is the spacing between the patches. While the last four scenarios with vegetation patches (2.7 cm x 21.6 cm) with *ʎ* = 5% and 10% along the side of the flume in the same section. In this arrangement, the *d/s* ratio varies from 0.05 to 0.20, depending on the vegetation plan. All the scenarios were performed under 0.060 m^3^/s flow with two conditions having with/without the tailgate.


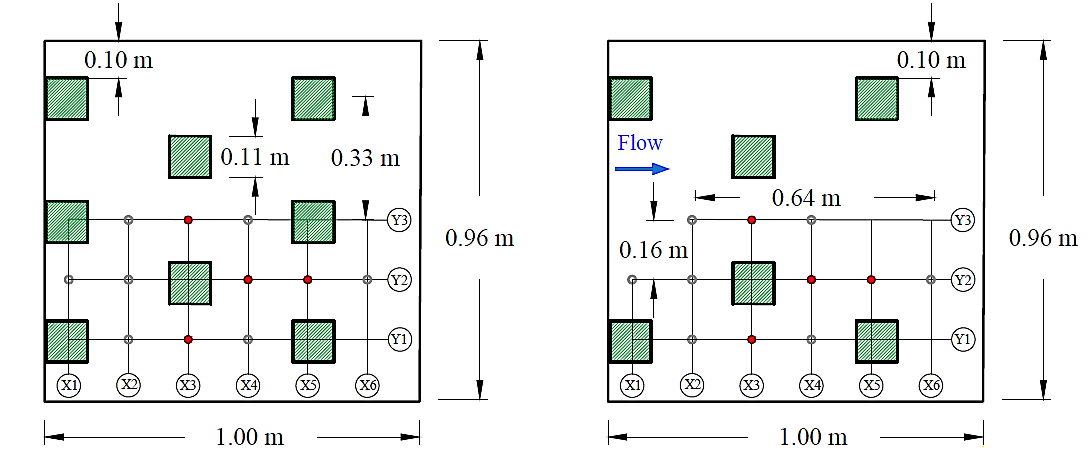

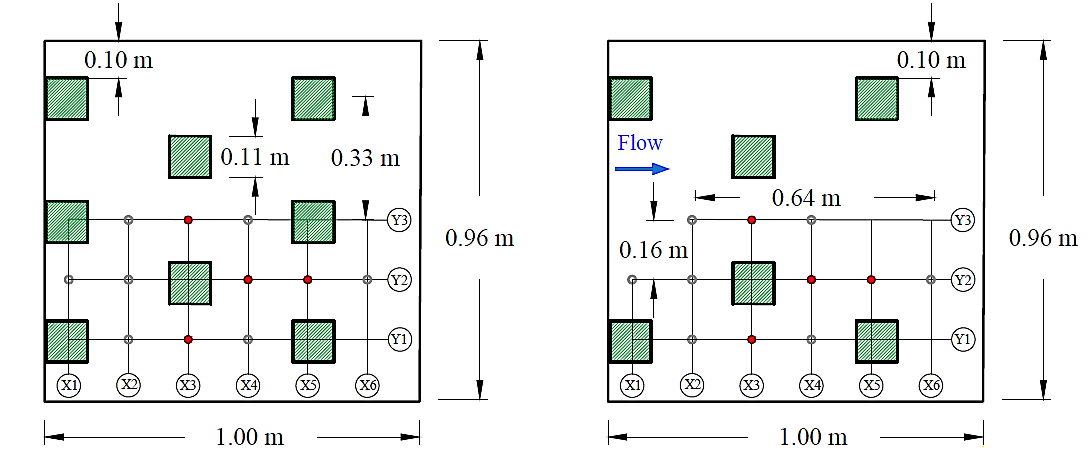


**(a)**

**(b)**


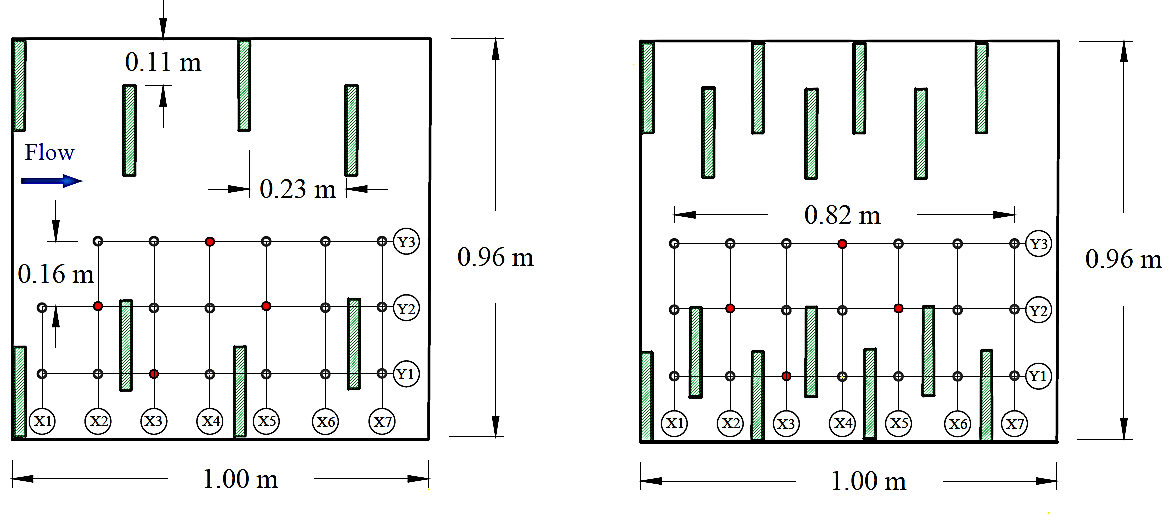

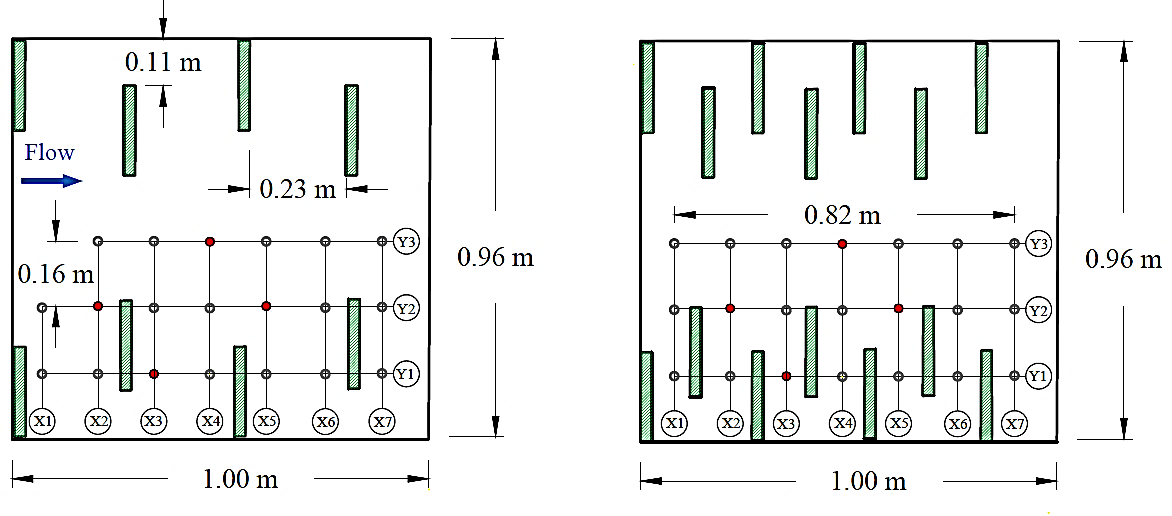


**(d)**

**(c)**

**Figure S1.** Plan view of the general measurement zone for experimental scenarios (a) B1 and B2, (λ = 7%) (b) B3 and B4 (λ =10%) with disperse vegetation, and (c) C1 and C2 (λ = 5%), (d) C3 and C4 with vegetation along banks (λ = 10%). The grid shows the detailed measurement zone and red circles show the location of hydraulic measuring stations.

*S1.3 Boulders scenarios*

For the boulder scenarios, natural, spherical-shaped boulders were used. The dimensions of the boulders were an equivalent size of *d* = 0.12 m in length and width, and *l*= 0.10 m in height. A combination of two different flow rates (*Q* = 0.060 m^3^/s and 0.075 m^3^/s) and four boulder concentrations (projected area/area of the measurement zone *λ* = 0, 3.4, 5.4, and 8.3%) were used to design six experimental scenarios (Figure S2). The increasing densities of boulders were used to show the effect of boulders and the induced changes in hydraulics on the retention and transport of the PS-MPs. The boulders were kept equal distances from one another at 0.72 meters apart, which is six times the boulder diameter and the *d/s* is equal to 0.16 in the longitudinal direction. This separation was to ensure there is little to no interaction between changes in the flow from one boulder to the next^2^.


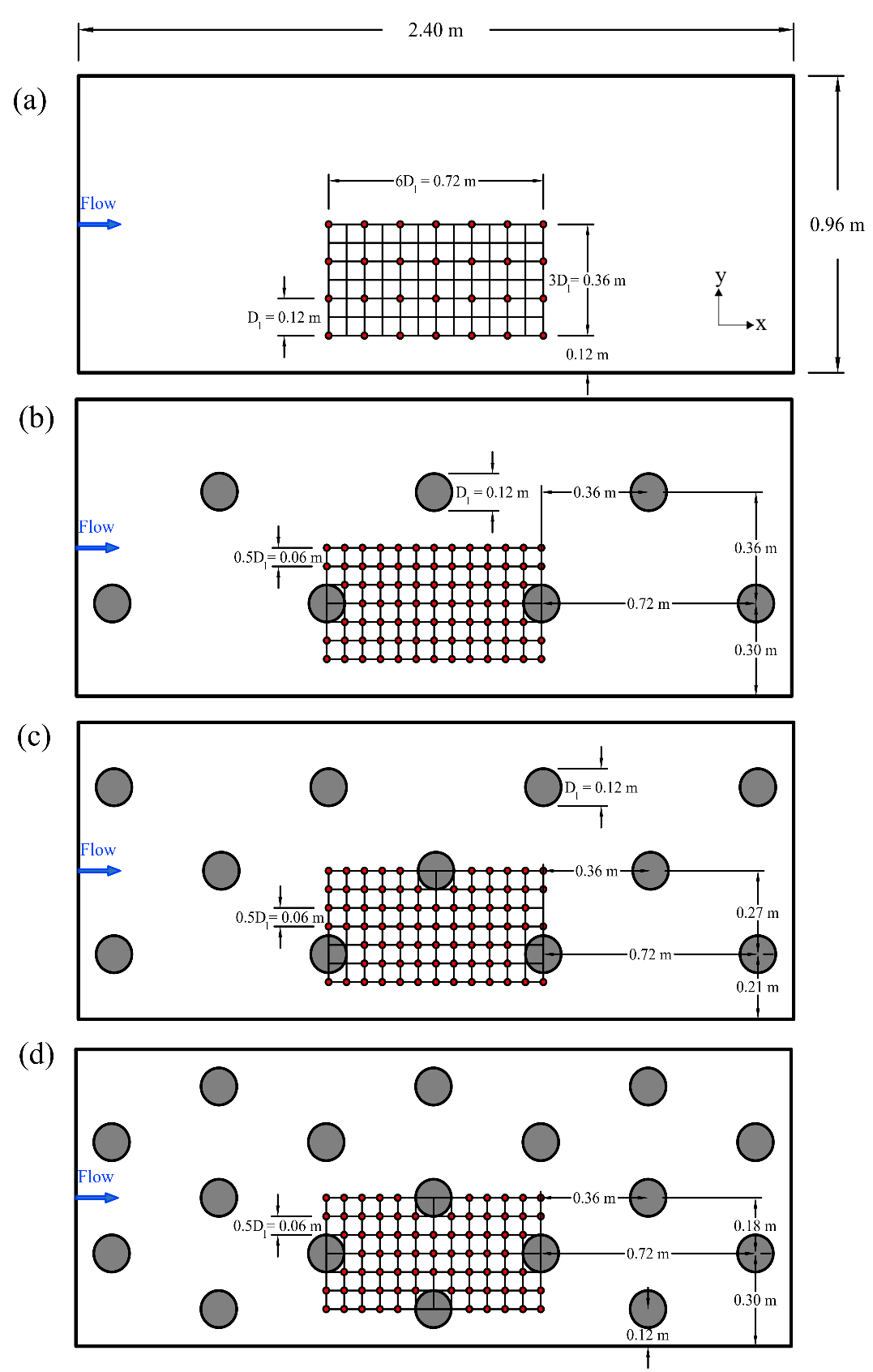


**Figure S2.** Scheme of the general measurement zone for experimental scenarios (a) A1, A2 and A3 (λ = 0%), (b) D1 and D2 (*λ* = 3.4%), (c) D3 and D4 (*λ* = 5.4%), and (d) D5 and D6 (*λ* = 8.3%). The grid shows the detailed measurement zone and red circles show the location of measuring stations adopted from Golpira et al. (2021).

**Reference**

1. Baptist, M. A flume experiment on sediment transport with flexible, submerged vegetation. In Int. workshop on riparian forest vegetated channels: hydraulic, morpho. and ecolo. asp, 20–22 (2003).
2. Golpira, A., Baki, M., Ghamry, H., Katopodis, C., Withers, J., and Minkoff, D. An experimental study, effects of boulder placement on hydraulic metrics of instream habitat complexity. *Scien. Rep*, **12**, 1-18 (2022).
